# Association of body mass index from childhood to mid-adulthood with health-related quality of life in mid-adulthood

**DOI:** 10.1007/s11136-023-03497-9

**Published:** 2023-09-05

**Authors:** Jing Tian, Leigh Blizzard, Julie A. Campbell, Seana Gall, Terence Dwyer, Alison Venn

**Affiliations:** 1 Menzies Institute for Medical Research, University of Tasmania, 17 Liverpool Street, Hobart, TAS 7000 Australia; 2 The George Institute for Global Health, University of Oxford, Wellington Square, Oxford, UK

**Keywords:** Body mass index, Trajectories, Quality of life, Health state utilities, Longitudinal studies

## Abstract

**Purpose:**

Most studies regarding the association of obesity with health-related quality of life (HRQoL) have assessed obesity at only one or two time points. We aimed to examine the associations of life course body mass index (BMI) from childhood with health-related quality of life (HRQoL) in mid-adulthood.

**Methods:**

Data were from a cohort study of Australian children (*n* = 2254, mean baseline age 12.0 (2.0) years in 1985, 46.8% male). Weight and height were measured at baseline and measured or self-reported on average 20, 25, and 30 years later. Age and sex-standardised BMI-z score was calculated at each time point. Physical and mental HRQoL and health state utilities (HSUs) were measured by SF-12 and SF-6D at the last adult follow-up. Linear regression was used to examine the associations adjusting for age, sex, and childhood health status.

**Results:**

Higher BMI-z score in childhood (*β*_adjusted_ − 1.39, 95% CI − 1.73 to − 1.05) and increasing BMI-z score from childhood to young adulthood (*β*_adjusted_ − 1.82, 95% CI − 2.17 to − 1.46) and from young to mid-adulthood (*β*_adjusted_ − 1.77, 95% CI − 2.28 to − 1.26) were associated with lower physical HRQoL in mid-adulthood. Similar results were found for mid-adulthood HSUs (*β*_adjusted_ ranged − 0.006 to − 0.014, all *P* < 0.05). Only increasing BMI-z score from young to mid-adulthood significantly related to poorer mental HRQoL (*β*_adjusted_ − 0.74, 95% CI − 1.29 to − 0.19) in mid-adulthood.

**Conclusion:**

High BMI from childhood to mid-adulthood had only modest associations with HRQoL and HSUs, with effects on physical HRQoL most apparent.

**Supplementary Information:**

The online version contains supplementary material available at 10.1007/s11136-023-03497-9.

## Plain English Summary

Literature regarding the association of obesity and health-related quality of life (HRQoL) is limited to studies assessing obesity at only one or two time points, ignoring the dynamic nature of weight over the life course. Using data from a large national cohort of school children in Australia, we aimed to assess the associations of body mass index (BMI, an indicator of obesity) from childhood to midlife with HRQoL and health state utilities (HSUs) in later life, over 30 years follow-up. We found that high BMI over the life course had only modest associations with HRQoL and HUSs in midlife, with effects on physical HRQoL most apparent.

## Introduction

Since 1975, the worldwide prevalence of overweight and obesity amongst children and adolescents has increased more than four-fold, from 4% in 1975 to 18% in 2016 [1]. Obesity tends to track from childhood to adulthood. Children and adolescents with overweight or obesity are at least twice as likely to be adults with overweight or obesity compared with their peers with a healthy weight [2, 3]; however, it is also known that around 90% of people with overweight or obesity in adulthood have a healthy weight in childhood [4], demonstrating the dynamic nature of weight over the life course.

Overweight and obesity are major risk factors of highly prevalent chronic diseases, such as cardiovascular disease (CVD)—the leading cause of morbidity and mortality around the world in 2016 [5]. These obesity-related diseases can take decades to develop, whereas the impacts of excess weight on health can be apparent in the short term through reduced health-related quality of life (HRQoL) [6]. HRQoL is a multi-dimensional concept that includes subjective evaluations of physical, mental, and social functioning influenced by a disease or health state.

Evidence from cross-sectional studies has demonstrated lower physical HRQoL in adults and children with overweight/obesity compared to their peers with normal weight, with a clear dose–response relationship across weight categories [7, 8]. A weaker or less consistent association was reported with mental HRQoL [7, 8]. In a recent systematic review of the longitudinal association between weight change and HRQoL in the general population, Hayes et al. [9] found a consistent, dose–response association between weight gain and lower physical HRQoL, whilst its association with mental HRQoL (except the subclass of vitality) was less consistent. Compared with weight gain, weight loss was less strongly associated with HRQoL amongst adults [9]. The relationships between weight change and HRQoL in children were found to mimic those in adults [9]. These results have deepened our understanding of the association between weight change and HRQoL; however, none of the studies included in the previous review or studies published later [10, 11] have covered a period long enough to encompass the life course from childhood to adulthood.

Health state utilities (HSUs) are an estimate of HRQoL and are important health economic metrics that assess the strength of preference for an individual’s health status relative to perfect health valued at 1.0 and death anchored at 0. Identification of people with lower HSUs helps to improve health resource allocation and better target health promotion activities and interventions. Evidence on the association of weight or weight change with HSUs has been mainly from cross-sectional studies [12, 13] or participants with specific health conditions (i.e. people with severe obesity who underwent bariatric surgery) [14] which are less readily generalisable to the general population. Research using longitudinal data to examine how weight impacts HUSs is scarce. Using multi-wave data from the Australian Longitudinal Study on Women’s Health, Kanesarajah and colleagues found that obesity was associated with lower mean HSUs across young, middle-age, and older cohorts of women [15]. This paper did not provide estimates of the effect on HSUs of weight change over the life course [15]. To the best of our knowledge, no study has explored the predictive association of weight trajectories with HSUs.

Using data from a population-based national cohort in Australia, we aimed to examine whether body mass index (BMI) from childhood to mid-adulthood was associated with mid-adulthood HRQoL and HSUs. We hypothesised that high and increasing BMI from childhood to mid-adulthood were associated with a lower HRQoL and HSUs in mid-adulthood.

## Methods

### Study design

A prospective cohort study.

### Participants

Participants were from the Childhood Determinants of Adult Health (CDAH) study, a 30-year follow-up of school children aged 7–15 years who participated in the 1985 Australian Schools Health and Fitness Survey (ASHFS) (*n* = 8498) [16]. A two-stage probability sampling framework was used to achieve a nationally representative sample: sampling of schools and sampling of boys and girls in each age group. Participants were followed up by completing questionnaires and attending clinics for physical measurements in 2004–2006 when aged 26–36 years (CDAH-1, *n* = 3998) and in 2014–2019 when aged 38–49 years (CDAH-3, *n* = 2177). The second follow-up (CDAH-2) was conducted in 2009–2011 where data were collected from 3038 participants (31–41 years) via telephone, mail or online survey.

The ASHFS was approved by the Directors of Education in each state and consent was obtained from children and parents. The Southern Tasmanian Health and Medical Ethics Committee approved the follow-up studies (CDAH-1, -2, and -3) and written informed consent was obtained from participants.

### Anthropometric measurements over the life course

Weight and height were objectively measured in childhood and either measured or self-reported in the three adult follow-ups. Subsamples of participants with both measured and self-reported weight and height in CDAH-1 (*n* = 1185) and CDAH-3 (*n* = 1236) were used to create adjusted self-reported measures in each follow-up [4, 17]. Specifically, measured weight and height were utilised to predict the difference between self-reported and measured weight and height. A linear regression model was used to obtain a correction factor that gave estimates of measured weight and height from self-reported values in CDAH-1 and CDAH-3, respectively [4, 17]. The calculated correction factors were then applied to participants who did not visit a study clinic in CDAH-1 and CDAH-3 and self-reported their weight and height to adjust for error. BMI (kg/m^2^) was derived from either clinic measured or adjusted self-reported weight and height as described above. Since weight was self-reported in CDAH-2, the correction factor calculated for CDAH-1 was used to estimate the adjusted weight values. BMI in CDAH-2 was calculated using measured height in CDAH-1 if it is available or adjusted height in CDAH-1 and adjusted weight in CDAH-2. In childhood, age- and sex-standardised BMI-z score was generated using the STATA igrowup package that implements the World Health Organisation Child Growth Standards and weight status was defined according to international age- and sex-specific cut-points [17, 18]. Adult age- and sex-standardised BMI-z score in each follow-up was generated using internal reference—CDAH participants in each adult visit.

### Assessment of health-related quality of life (HRQoL)

The 12-item Short Form Health Survey (SF-12) version 2 [19] was used to assess HRQoL in the last adult follow-up, i.e. either CDAH-2 or -3. It has eight health domains with each scored from 0 (worst possible health) to 100 (best possible health): physical functioning (2 items), role limitations due to physical problems (2 items), bodily pain (1 item), general health perceptions (1 item), vitality (1 item), social functioning (1 item), role limitations due to emotional problems (2 items), and mental health (2 items). These eight domains were summarised into the physical component summary (PCS) and the mental component summary (MCS) by means of factor analyses. These scores were calculated based on US population normative values with a mean of 50 and a standard deviation of 10. Higher scores denote better HRQoL. There is debate about the minimal clinically important difference (MCID) in HRQoL scores, but to be consistent with our previous work [20], we used 5 points as the threshold.

### Assessment of health state utilities (HSUs)

In the last adult follow-up (CDAH-2/-3), HSUs were generated from the participant-reported responses to the SF-12 version 2 (seven items) via the SF-6D, a multi-attribute utility instrument algorithm specifically for the SF-12 [21]. These HSUs were based on the United Kingdom (UK) value set in the absence of an Australian value set for the SF-12-based SF-6D [21]. The HSUs value generated by the SF-6D’s algorithm ranges from 1.00 (best HRQoL state or full health) to 0.30 (worst HRQoL state) [21]. The SF-6D has an equal preponderance to physical and psychosocial health covering six domains including physical function, role limitation, social function, bodily pain, mental health, and vitality. HSUs are used as an input metric for the ‘quality adjusted life years’ component in cost-utility analysis that assists healthcare decision makers with resource allocation decisions [22]. HSUs are also increasingly being used for clinical management and predictors of health [23]. Lower HSUs represent reduced HRQoL, which leads to increased health service utilisation and expenditure. To assess the significance of the HSUs variation across different patterns of BMI from childhood to mid-adulthood, we adopted 0.04 as the threshold of MCID [24].

### Potential confounders

Children and adolescents aged 9–15 years completed questionnaires on demographic and behaviours in small groups of four, under supervision from a study data collector. Children under 9 years of age were deemed too young to complete the questionnaires reliably. Childhood variables included sex, area-level disadvantage (categorised in quarters: high, medium high, medium low, and low) [25, 26], language spoken at home (English or other), urban–rural status, smoking experimentation (none, a few puffs, and have smoked fewer than 10 cigarettes or more than 10 cigarettes in my life) [26], alcohol consumption (never, < once per week, and ≥ once per week) [27], breakfast consumption (yes/no) [27], total physical activity (PA) (h/week) [27], and self-rated health status (very good, good, average/poor/very poor). Highest parental education (university education, vocational training, and high school or less) [28] and highest parental occupation (managers/professionals/white collar, and blue collar/unpaid/unemployed) [26] were retrospectively reported by participants at CDAH-1. Age at CDAH-2/-3 was also considered as a potential confounder.

### Statistical analysis

Mean [standard deviation (SD)] or median [interquartile range (IQR)] for continuous variables and % (n) for categorical variables were used to describe the characteristics of participants. Comparisons between participants and those lost to follow-up were performed using t-test for continuous variables with normal distribution and Kruskal–Wallis test otherwise. Chi-square tests were used for the comparison of categorical variables. Linear regression models were used to examine the associations of BMI-z scores from childhood through young to mid-adulthood with HRQoL and HSUs in mid-adulthood. Values of HRQoL and HSUs were transformed (e.g. by taking logarithms) to remove skewness and make the distribution of their residuals more normal-like. All estimates were reported after back transformation to the original scale. BMI was a continuous time-varying exposure with measurements made in childhood, young adulthood, and mid-adulthood. The covariates included in the regression models were formed from the BMI-z scores at the three time-points, and from their sums and differences. The use of the sum of time-specific values is consistent with the hypothesis in life course epidemiology that risk is conferred by the “accumulation” [29, 30] or “lifetime effect” [31] of exposure to a study factor. The use of differences between time-specific values would be warranted if it is “mobility” [29] or “change” [30, 31] in exposure that confers risk. Including the covariate for a single time-specific BMI z score (the values in childhood, for example) signals that time point represents a “critical period” [29], whilst including a mix of the covariates for time-specific BMI-z scores, their sums and differences, and their interactions allows more complex patterns to be modelled. The selection of covariates to be retained was based on tests of linear contrasts.

Potential confounders were included in the adjustment if they were causally related to the outcome according to prior knowledge, unbalanced between the exposure groups and caused a change of 10% or more in the estimated coefficients when included in a given regression model.

To examine the effects of loss to follow-up on the results, we compared the baseline (childhood) data of participants and those lost to follow-up, and performed sensitivity analyses using inverse probability weighting (IPW) with multiple imputation (MI) [32]. This sensitivity analyses still used the same sample but weighted them to make more similar to childhood sample, which is nationally representative of school children aged 7–15 years in 1985*.* Childhood variables which predicted missingness were used in the calculation of weights in IPW, including childhood age, sex, school type, area-level disadvantage, urban–rural status, weight status, anthropometric measures (height z score, weight z score, arm girth z score, waist girth z score, and hip girth z score), fitness (sit and reach z score, sit-ups z score, standing long jump z score, time for 1.6 km run z score, and time for 50 m run z score), school enjoyment, learner self-concept good at schoolwork, school assessed scholastic ability, self-reported health status, passive smoking, alcohol experimentation, dietary guidelines index (DGI), and physical activity (PA). These variables were nearly complete at baseline, with a completion rate ranging from 74.1 to 99.9%. MI was employed to impute missing data for these variables using their available information and three complete variables in childhood including age, sex, and school type.

Subgroup analysis was performed to investigate whether participants who had the highest increase in BMI from childhood to mid-adulthood have the lowest scores of HRQoL and HSU in mid-adulthood. Participants were defined as having the highest increase in BMI over the life course if their increase in BMI-z score was persistently more than one standard deviation (SD) from ASHFS to CDAH-1 and from CDAH-1 to CDAH-2/-3 (highest BMI increase group, *n* = 40). Participants were defined as the lower BMI change group if their BMI-z score and change in BMI-z score over the life course were within one SD (*n* = 1029). A two-tailed P value less than 0.05 was considered statistically significant. All analyses were performed with STATA software, version 16.1 (Stata Corp, College Station, Texas 77845 USA).

## Results

**Fig. 1:**
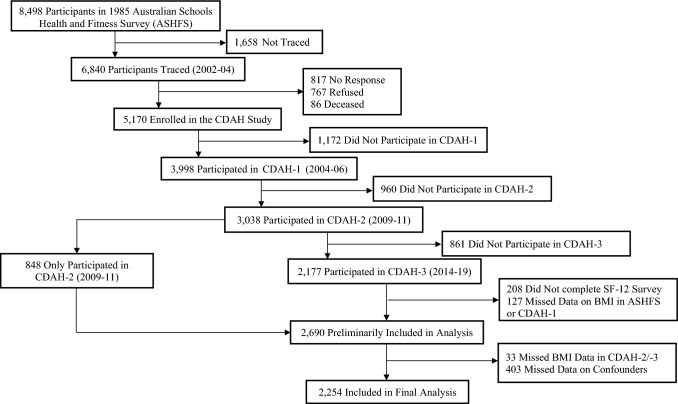
Flow chart of selected participants in Childhood Determinants of Adult Health (CDAH) study, Australia, 1985–2019

Analyses were restricted to participants who had complete data on the outcomes, exposures, and confounders (*n* = 2254, Fig. 1). The mean (SD) follow-up length was 30.3 (3.6) years. The sociodemographic characteristics of participants in childhood and mid-adulthood are presented in Table 1. In childhood, the mean age was 12.0 years and 46.8% were males. Most (91%) children had a normal weight, 7.9% were affected by overweight and 1.2% were affected by obesity. Most (80.7%) children reported very good and good health. In mid-adulthood, the mean age was 42.3 years, and the mean BMI was 27.3 kg/m^2^. On average, the physical HRQoL was 51.1 in mid-adulthood, the mental HRQoL was 50.1, and the HSU was 0.761.

**Table 1:** Sociodemographic characteristics of participants in childhood and mid-adulthood

Characteristics	*n*	Total participants
*Childhood*		
Age (years), Mean (SD)	2254	12.0 (2.0)
Male, % (*n*)	2254	46.8 (1055)
Body mass index (kg/m^2^), Mean (SD)	2254	18.6 (2.8)
Body mass index *z* score, Median (IQR)	2254	0.14 (− 0.48, 0.74)
Weight status, % (*n*)	2254	
Normal		91.0 (2050)
Overweight		7.9 (178)
Obese		1.2 (26)
Highest parental education, % (*n*)	1646	
Any university education		26.9 (442)
Vocational training		33.0 (543)
High school only		40.2 (661)
Highest parental occupation, % (*n*)	1677	
Professional/white collar		74.5 (1250)
Blue collar/unpaid/unemployed		25.5 (427)
Area-level SEP, % (*n*)	2208	
High		26.3 (581)
Medium–high		27.5 (608)
Medium–low		38.9 (858)
Low		7.3 (161)
English at home, % (*n*)	2249	89.7 (2017)
Urban–rural status, % (*n*)	2227	
Urban		78.3 (1744)
Rural		21.7 (483)
Physical activity (h/w), Median (IQR)	2254	5.5 (3.2, 9.4)
Weekly physical activity*, % (*n*)	2227	
Yes		44.0 (979)
No		56.0 (1248)
Eat breakfast, % (*n*)	2249	85.8 (1929)
Smoking experimentation, % (*n*)	2248	
Never smoked		55.0 (1236)
Few puffs		24.6 (553)
Smokes		20.4 (459)
Alcohol, % (*n*)	2251	
Don’t drink		67.0 (1509)
< Once/week		26.3 (591)
1 or more/week		6.7 (151)
Self-rated health, % (*n*)	2254	
Very good		37.1 (836)
Good		43.6 (982)
Average/poor/very poor		19.3 (436)
*Mid-adulthood*		
Age (years), Mean (SD)	2254	42.3 (4.2)
Body mass index (kg/m^2^), Mean (SD)	2254	27.4 (5.6)
Body mass index *z* score, Median (IQR)	2254	− 0.18 (− 0.66, 0.51)
Physical component summary, Mean (SD)	2254	51.1 (8.0)
Mental component summary, Mean (SD)	2254	50.1 (9.3)
Health state utilities, Mean (SD)	2254	0.761 (0.128)

*IQR* interquartile range, *SD* standard deviation, *SES* socioeconomic position

*Yes was defined as having exercise or activity 3 or 4 times in most weeks which makes huff and puff and lasts at least 30 minutes each time

Using baseline (childhood) characteristics (Table S1), compared with those lost to follow-up, participants were older, more often female, and more likely to have normal weight, and higher socioeconomic position (based on maternal education, urban–rural status, and area-level disadvantage), better self-rated health, healthier lifestyles (including PA, DGI, and alcohol experimentation), and more favourable fitness and school-related measures (including school enjoyment, learner self-concept good at schoolwork, and school assessed scholastic ability).

Table 2 presents the longitudinal association of BMI-z score from childhood to mid-adulthood with HRQoL and HSUs in mid-adulthood. According to estimated coefficients for BMI-z score in childhood, young and mid-adulthood (Table S2), the “accumulation” or “lifetime” effect model best fit the associations of life course BMI with physical HRQoL and HSUs in mid-adulthood, and the “change” or “mobility” model best fit the association with mental HRQoL in mid-adulthood. In unadjusted models (Table 2), the “accumulation” or “lifetime” effect model showed that higher BMI-z score in childhood and subsequent increases in BMI-z score from childhood through young to mid-adulthood were significantly associated with lower physical HRQoL and HSUs in mid-adulthood. On average, one unit increase in childhood BMI-z score was associated with a lower score of 1.49 in physical HRQoL and a lower score of 0.009 in HSUs in mid-adulthood, and one unit increase in BMI-z score from childhood to young adulthood and from young to mid-adulthood was related to 1.89 and 1.86 lower scores in physical HRQoL and 0.008 and 0.016 lower scores in HSUs in mid-adulthood, respectively. Adjustment for age at mid-adulthood, sex, and self-reported health status at baseline slightly attenuated the associations but they remained statistically significant (Model 1). The association between BMI-z score from childhood to mid-adulthood and mental HRQoL in mid-adulthood was weak, with only an increase in BMI-z score from young to mid-adulthood related to a lower score of 0.82 and 0.74 in mental HRQoL in mid-adulthood in unadjusted and multivariable models. There was no evidence supporting an association between childhood BMI-z score and mental HRQoL in mid-adulthood. The full regression outputs including all independent variable are presented in Table S3.

**Table 2:** The longitudinal association of BMI from childhood to mid-adulthood with health-related quality of life and health state utilities in mid-adulthood, before and after applying inverse probability weighting with multiple imputation^*^

BMI-z score (*N* = 2254)	Unadjusted model	Model 1^#^	Model 1^#^ + IPW & MI
	*β* (95% CI)	*β* (95% CI)	*β* (95% CI)
Physical component summary			
In ASHFS (baseline)	− 1.49 (− 1.84, − 1.14)	− 1.39 (− 1.73, − 1.05)	− 1.43 (− 1.81, − 1.05)
Change from ASHFS to CDAH-1	− 1.89 (− 2.26, − 1.53)	− 1.82 (− 2.17, − 1.46)	− 1.90 (− 2.31, − 1.49)
Change from CDAH-1 to CDAH-2/-3	− 1.86 (− 2.39, − 1.34)	− 1.77 (− 2.28, − 1.26)	− 1.78 (− 2.37, − 1.20)
Mental component summary			
In ASHFS (baseline)			
Change from ASHFS to CDAH-1			
Change from CDAH-1 to CDAH-2/-3	− 0.82 (− 1.40, − 0.24)	− 0.74 (− 1.29, − 0.19)	− 1.05 (− 1.94, − 0.15)
Health state utilities			
In ASHFS (baseline)	− 0.009 (− 0.015, − 0.003)	− 0.007 (− 0.014, − 0.001)	− 0.008 (− 0.015, − 0.002)
Change from ASHFS to CDAH-1	− 0.008 (− 0.014, − 0.001)	− 0.006 (− 0.013, 0)	− 0.006 (− 0.013, 0.001)
Change from CDAH-1 to CDAH-2/-3	− 0.016 (− 0.026, − 0.007)	− 0.014 (− 0.023, − 0.005)	− 0.015 (− 0.026, − 0.005)

^*^Age, sex, and school type at baseline were used to impute missing data; age, sex, school-type, socio-economic position, weight status, anthropometric measures (height z score, weight z score, arm girth z score, waist girth z score and hip girth z score), fitness (sit and reach z score, sit-ups z score, standing long jump z score, time for 1.6 km run z score, and time for 50 m run z score), school enjoyment, learner self-concepted good at schoolwork, school assessed scholastic ability, self-reported health status, passive smoking, alcohol experimentation, dietary guidelines index, and total physical activity at baseline were used to determine the weights

*ASHFS* Australian Schools Health and Fitness Survey, *BMI* body mass index, *CDAH* Childhood Determinants of Adult Health study, *CI* confidence interval, *HRQoL* health-related quality of life, *IPW* inverse probability weighting, *MI* multiple imputation

^#^Adjusted for mid-adulthood age, sex, and self-reported health status at baseline

On average, the adjusted differences in physical HRQoL, mental HRQoL, and HSUs in mid-adulthood between those who consistently stayed in the position of mean BMI for their age and sex from childhood through young to mid-adulthood, and those who stayed in the position of one SD above mean BMI for their age and sex at these three time points, were − 1.45 (physical HRQoL), − 0.13 (mental HRQoL), and − 0.007 (HSUs) (Table S2). These values are much smaller than the recommended MCID for HRQoL (score of 5) and HSUs (score of 0.04), with effects on physical HRQoL most apparent.

Sensitivity analyses using IPW with MI to account for loss to follow-up showed similar trends and slightly stronger associations (Table 2).

The distribution of HRQoL and HSUs in mid-adulthood by BMI-z score from childhood to mid-adulthood is shown in Table S4. Compared to participants in the lower BMI change group, those in the highest BMI increase group reported statistically significant lower scores of physical HRQoL, mental HRQoL, and HSUs in mid-adulthood. These differences persisted after adjustment for mid-adulthood age, sex, and self-reported health status at baseline (Table S5). On average, only the adjusted difference for HSUs in mid-adulthood was greater than the MCID (0.04 for HSUs).

## Discussion

To our knowledge, this is the first study to have utilised BMI data from three time points spanning childhood and adulthood to examine associations with later life HRQoL and HSUs. In this population-based prospective study of Australian children with 30 years follow-up, we found that childhood BMI and BMI gain from childhood through young to mid-adulthood were statistically negatively associated with physical HRQoL and HSUs in mid-adulthood. Only BMI gain from young to mid-adulthood significantly related to poorer mental HRQoL in mid-adulthood. Whilst these findings suggested adverse impacts of high BMI and weight gain on physical and mental well-being, the strength of these associations was weak.

High BMI in childhood was found to predict poor physical HRQoL in mid-adulthood. A systematic review and meta-analysis in children and adolescents has shown that children with obesity had significantly lower physical functioning compared with their lean counterparts [33]. Its pooled analyses also showed a strong inverse linear relationship between BMI and physical functioning score [33]. It was expected that these physical impairments could track, accumulate, and carry negative implications into adulthood. Our finding was contrary to the only comparable study from Canada, in which no significant association was found between continuous BMI or weight status for youth aged 7–18 years and physical HRQoL 22 years later [34]. A possible explanation for the difference is the various approaches for analyses. We used a novel analytical technique which simultaneously takes into account childhood BMI and subsequent changes, whilst the Canadian study only considered the childhood BMI and did not tease out the change between two time points, as noted in their discussion [34]. Future research including multiple measurements of BMI from childhood is needed to confirm our finding.

Apart from childhood BMI-z score, we found increase in BMI-z score from childhood to adulthood also predicted poorer HRQoL in mid-adulthood. The persons most likely to have poor physical HRQoL were those who had high BMI-z score in childhood without subsequent declines in BMI-z score and those who had low BMI-z score in childhood but with large subsequent increases in BMI-z score. Prior evidence on the predictive association of weight change with later life physical HRQoL was inconsistent. A recent systematic review found that studies which analysed weight change as a categorical variable support our finding by consistently showing that weight gain predicted lower physical HRQoL or physical subdomains in both children and adults [9] but no significant association was found between weight change as a continuous variable and physical HRQoL at follow-up. Possible explanations include the shorter follow-up length in studies analysing weight change as a continuous variable than as a categorical variable (2–5 years versus 5–8 years), different indicators of adiposity (abdominal versus general), and the use of different scales for HRQoL measurement (KIDSSCREEN-27 and Impact of weight on Quality of Life-Kids versus Paediatric Quality of Life Inventory) [9].

No significant association was found between childhood BMI and mental HRQoL in mid-adulthood. This finding was consistent with most previous literature in children [9, 35] but not all [34, 36]. In a 22-year follow-up study of a small group of participants from the 1981 Canada Fitness Survey, Herman and colleagues unexpectedly found a positive association of youth overweight/obesity with adult mental HRQoL and its subdomains [34], though only in females [36]. Although the results were consistent for categorical weight status and continuous BMI, the study suffered from low statistical power (only one obese child at baseline) and high attrition [34, 36], so results should be interpreted cautiously. Using the computerised Composite International Diagnostic Interview (CIDI-Auto, Version 2.1), our previous work found that overweight/obesity [37] or high BMI [38] in early life (childhood and young adulthood) was associated with an increased risk of mood disorder in later life (young adulthood and mid-adulthood). Our current finding seems contradictory to this but direct comparison of these results is inappropriate, mainly because of the different outcome measures: SF-12 measured mental HRQoL during the past 4 weeks, whereas mood disorder in the past 12 months was assessed by the CIDI, a computerised diagnostic instrument, in our previous work. In addition, the studied time periods are different: an average follow-up of 30 years in the current study versus 20 and 5 years in prior work.

We also found an increase in BMI from young to mid-adulthood was associated with lower mental HRQoL in mid-adulthood. The reasons why change in BMI-z score from young to mid-adulthood appears more important for mental HRQoL than the change in BMI-z score from childhood to young adulthood are unclear. However, many major life-stage transitions occur in this period as young people complete education and training and as their family, work and financial responsibilities increase. These transitions are often accompanied by changes in weight-related lifestyle behaviours including smoking [39], diet [40], physical activity and sedentary behaviours [41, 42], and mental health as well [43–45]. Results for the predictive association between life course BMI and HSUs in mid-adulthood were generally consistent with the findings for physical HRQoL. Although a few studies have examined the association of childhood BMI and HSUs and produced mixed findings, a direct comparison cannot be made with our findings because those studies were either cross-sectional [12, 13, 46] or repeated cross-sectional [15] in study design. Nevertheless many co-morbid conditions like metabolic, cardiovascular, orthopaedic, neurological, hepatic, pulmonary, and renal disorders have been seen in association with childhood obesity [47], including at a young age [48], and these might be expected to associate with HSUs. Understanding the complex relationship how and why BMI and HRQoL interact over the life course may require use of qualitative and quantitative methods in future work.

Although high and increasing BMI-z score from childhood was found to be statistically significantly associated with later life HRQoL and HSUs, it is also important to consider the strength of these associations. As described in the results, the magnitude of the adjusted differences in physical HRQoL, mental HRQoL, and HSUs in mid-adulthood between those who consistently stayed in the position of mean BMI for their age and sex from childhood through young to mid-adulthood, and those who stayed in the position of one SD of BMI for their age and sex at these three time points are much smaller than the recommended MCID for HRQoL (score of 5) and HSUs (score of 0.04), indicating these associations were weak even though they were statistically significant, with effects on physical HRQoL most apparent. However, it is also worthwhile noting that participants who had the highest increase in BMI from childhood to mid-adulthood (highest BMI increase group) reported statistically significant and clinically important lower score of HSUs in mid-adulthood than those who had lower BMI change over the life course, indicating that this is a high-risk group that may benefit from health promotion or weight management intervention.

Some limitations should be considered in the interpretation of our results. First, although the baseline sample was nationally representative of Australian school children aged 7–15 years, those who were followed-up through to mid-adulthood showed differences on some socio-demographics compared with those loss to follow-up or the Australian general population of adults aged 35–44 years. Applying the inverse probability weighting after multiple imputation to account for the differences showed similar results, suggesting that bias due to attrition is minimal. Of note, although the differences at follow up in demographic structure do not seem likely to have been responsible for our findings, it is possible that unmeasured determinants could have introduced bias. Second, self-reported weight might underestimate the actual weight and self-reported height might overestimated the actual height. However, a correction factor has been calculated and used to reduce the potential error. In addition, changes in age- and sex-standardised BMI-z score between two adjacent visits were used in analyses. Previous data has revealed that the discrepancy between weight change based on self-reported versus measured weights was minor [49]. Third, although self-reported health status at baseline was adjusted in the key model, we didn’t have the full instrument for HRQoL measurement at baseline and therefore could not elucidate the possible impact of adjusting for baseline HRQoL on results.

Strengths of this study include a large national sample of children and adolescents, a long-term follow-up from childhood (mean 30 years), BMI measures from multiple time-points (3 or 4), a prospective design and the use of a novel analytical technique and advanced methodology to account for loss to follow-up. In addition, this is the first study to examine the relationships of life course BMI from childhood with HRQoL and HSUs in mid-adulthood.

To sum up, in this population-based prospective cohort of Australian children with 30 years follow-up, high and increasing BMI from childhood to mid-adulthood had only modest associations with HRQoL and HSUs in mid-adulthood, with effects on physical HRQoL most apparent.

### Supplementary Information

Below is the link to the electronic supplementary material.

Supplementary file1 (DOCX 37 KB)

## Data Availability

The datasets used and/or analysed during the current study are available from the corresponding author on reasonable request.
