# Supplementary material for: Association of body mass index from childhood to mid-adulthood with health-related quality of life in mid-adulthood
Source: Qual Life Res. 2023 Sep 5;32(12):3349–58. doi: 10.1007/s11136-023-03497-9 (PMC10624736; doi:10.1007/s11136-023-03497-9)
Supplement: Supplementary file 1 — Supplementary file1 (DOCX 37 KB) [file 11136_2023_3497_MOESM1_ESM.docx]

**Article title:** Association of body mass index from childhood to mid-adulthood with health-related quality of life in mid-adulthood.

**Journal name:** Quality of Life Research.

**Authors’ names:** Jing Tian, Leigh Blizzard, Julie Campbell, Seana Gall, Terence Dwyer, Alison Venn.

**Affiliation and e-mail address of the corresponding author**: Jing Tian ^*^, Menzies Institute for Medical Research, University of Tasmania, 17 Liverpool Street, Hobart, Tasmania, 7000, Australia; Phone: +61 3 6226 7700; Fax: +61 3 6226 7704; Email: J.Tian@utas.edu.au

**Table S1** Childhood characteristics at baseline of participants assessed and not assessed in adulthood, Childhood Determinants of Adult Health Study, Australia, 1985-2019^*^

| **Characteristics** | **Participants (n=2,254)** | **Loss to follow-up (n=** **6,244)** | **P-value** |
| --- | --- | --- | --- |
| Age (years), Mean (SD) | 12.0 (2.0) | 10.5 (2.6) | **<0.001** |
| Male, % (n) | 46.8 (1,055) | 52.1 (3,252) | **<0.001** |
| Weight status, % (n) |  |  | **<0.001** |
| Normal | 91.0 (2,050) | 87.2 (5,440) |  |
| Overweight | 7.9 (178) | 11.0 (683) |  |
| Obese | 1.2 (26) | 1.8 (113) |  |
| Area-level disadvantage, % (n) |  |  | **<0.001** |
| High | 26.3 (581) | 22.2 (909) |  |
| Medium-high | 27.5 (608) | 29.1 (1,192) |  |
| Medium-low | 38.9 (858) | 38.4 (1,569) |  |
| Low | 7.3 (161) | 10.3 (421) |  |
| Urban-rural status, % (n) |  |  | **<0.001** |
| Urban | 78.3 (1,744) | 82.6 (3,322) |  |
| Rural | 21.7 (483) | 17.4 (701) |  |
| Highest paternal education, % (n) |  |  | 0.364 |
| Any university education | 26.9 (442) | 23.2 (259) |  |
| Vocation training | 33.0 (543) | 32.7 (365) |  |
| High school only | 40.2 (661) | 44.1 (493) |  |
| Highest maternal education, % (n) |  |  | **0.004** |
| Any university education | 16.1 (260) | 19.5 (227) |  |
| Vocation training | 17.8 (287) | 20.4 (238) |  |
| High school only | 66.2 (1,070) | 60.1 (700) |  |
| Physical Activity (mins/wk), Median (IQR) | 330.0 (190.0, 562.0) | 320.0 (179.0, 560.0) | 0.135 |
| Smoking experimentation, % (n) |  |  | 0.233 |
| Never smoked | 55.0 (1,236) | 55.7 (2,256) |  |
| Few puffs | 24.6 (553) | 22.8 (924) |  |
| Smokes | 20.4 (459) | 21.5 (871) |  |
| Parental smoking in childhood |  |  | **<0.001** |
| None | 57.5 (1,289) | 46.2 (1,864) |  |
| One parent | 29.3 (657) | 35.4 (1,429) |  |
| Both parents | 13.1 (294) | 18.4 (744) |  |
| Alcohol, % (n) |  |  | **0.017** |
| Don’t drink | 67.0 (1,509) | 67.2 (2,787) |  |
| < Once/week | 26.3 (591) | 24.3 (1,007) |  |
| 1 or more days/week | 6.7 (151) | 8.5 (352) |  |
| Dietary guidelines index, Mean (SD) | 45.2 (12.1) | 44.2 (12.0) | **0.005** |
| Self-rated health, % (n) |  |  | **0.036** |
| Very good/good | 37.1 (836) | 34.7 (1,439) |  |
| Average | 43.6 (982) | 43.6 (1,808) |  |
| Poor/very poor | 19.3 (436) | 21.8 (905) |  |
| School type, % (n) |  |  | **0.001** |
| Primary | 72.7 (1,638) | 75.9 (4,737) |  |
| Secondary | 20.8 (468) | 19.3 (1,205) |  |
| Primary and secondary | 6.6 (148) | 4.8 (302) |  |
| School enjoyment ^*^, % (n) |  |  | **<0.001** |
| All of the time | 9.6 (216) | 11.9 (496) |  |
| Most of the time | 38.9 (877) | 33.6 (1,396) |  |
| Some of the time | 40.6 (916) | 38.8 (1,612) |  |
| A little of the time | 7.2 (162) | 8.6 (356) |  |
| None of the time | 3.7 (83) | 7.1 (293) |  |
| Learner self-concepted good at schoolwork, % (n) |  |  | **<0.001** |
| Better than other | 27.9 (627) | 21.9 (906) |  |
| Same as others | 64.8 (1,458) | 67.0 (2,779) |  |
| Worse than others | 7.4 (166) | 11.1 (462) |  |
| School assessed scholastic ability ^*^, % (n) |  |  | **<0.001** |
| Excellent | 12.2 (259) | 8.3 (484) |  |
| Above average | 33.3 (706) | 25.8 (1,505) |  |
| Average | 41.0 (868) | 41.3 (2,411) |  |
| Below average | 11.7 (247) | 18.6 (1,089) |  |
| Poor | 1.8 (39) | 6.0 (353) |  |
| Height z score, Mean (SD) | 0.05 (0.98) | -0.02 (1.01) | **0.006** |
| Weight z score, Mean (SD) | -0.04 (0.94) | 0.02 (1.02) | **0.012** |
| Arm girth z score, Mean (SD) | -0.07 (0.97) | 0.03 (1.01) | **<0.001** |
| Waist girth z score, Mean (SD) | -0.08 (0.93) | 0.03 (1.04) | **<0.001** |
| Hip girth z score, Mean (SD) | -0.05 (0.96) | 0.02 (1.03) | **0.004** |
| Sit and reach z score, Mean (SD) | 0.05 (1.01) | -0.01 (1.00) | **0.008** |
| Number of sit-ups in 5 minutes z score, Mean (SD) | 0.14 (1.03) | -0.05 (0.99) | **<0.001** |
| Standing long jump z score, Mean (SD) | 0.01 (0.94) | -0.11 (0.95) | **<0.001** |
| Time for 1.6 km run z score, Mean (SD) | 0.20 (0.68) | 0.30 (0.76) | **<0.001** |
| Time for 50m run z score, Mean (SD) | 0.04 (0.61) | 0.11 (0.61) | **<0.001** |

BMI: body mass index; IQR: interquartile range; SD: standard deviation.

^*^ Sample size varied because of missing data (range, 1,101-6,242 for the group of loss to follow-up and 1,568-2,253 for the group of participants).

**Table S2** Estimated coefficients for the association of BMI-z score in childhood, young and mid-adulthood to mid-adulthood with health-related quality of life and health state utilities in mid-adulthood

| **BMI-z score (N=2,254)** | **Unadjusted model** |  | **Model 1*** |
| --- | --- | --- | --- |
|  | **β (95% CI)** |  | **β (95% CI)** |
| **Physical component summary** |  |  |  |
| ASHFS | **0.39 (0.06, 0.73)** |  | **0.40 (0.07, 0.74)** |
| CDAH-1 | -0.01 (-0.54, 0.51) |  | -0.05 (-0.60, 0.47) |
| CDAH-2/-3 | **-1.87 (-2.40, -1.35)** |  | **-1.80 (-2.32, -1.28)** |
| **Mental component summary** |  |  |  |
| ASHFS | -0.19 (-0.60, 0.22) |  | -0.15 (-0.55, 0.25) |
| CDAH-1 | **0.83 (0.22, 1.44)** |  | **0.78 (0.19, 1.38)** |
| CDAH-2/-3 | **-0.83 (-1.43, -0.24)** |  | **-0.76 (-1.34, -0.18)** |
| **Health state utilities** |  |  |  |
| ASHFS | -0.001 (-0.008, 0.005) |  | -0.001 (-0.008, 0.005) |
| CDAH-1 | 0.009 (-0.002, 0.019) |  | 0.008 (-0.002, 0.017) |
| CDAH-2/-3 | **-0.016 (-0.026, -0.007)** |  | **-0.014 (-0.023, -0.005)** |

ASHFS, Australian Schools Health and Fitness Survey; BMI: body mass index; CDAH, Childhood Determinants of Adult Health study; CI: confidence interval.

^*^Adjusted for mid-adulthood age, sex and self-reported health status at baseline.

Values in bold are statistically significant results.

**Table S3** Full regression outputs for the longitudinal association of BMI from childhood to mid-adulthood with health-related quality of life and health state utilities in mid-adulthood

| **Independent variables (N=2,254)** | **Model 1** | **P-value** |
| --- | --- | --- |
|  | **β (95% CI)** |  |
| **Physical component summary** |  |  |
| BMI-z score in ASHFS | **-1.39 (-1.73, -1.05)** | **<0.001** |
| Change in BMI-z score from ASHFS to CDAH-1 | **-1.82 (-2.17, -1.46)** | **<0.001** |
| Change in BMI-z score from CDAH-1 to CDAH-2/-3 | **-1.77 (-2.28, -1.26)** | **<0.001** |
| Age in CDAH-2/-3 (mid-adulthood), year | **0.37 (0.31, 0.44)** | **<0.001** |
| Gender |  |  |
| Male | Ref |  |
| Female | 0.23 (-0.30, 0.75) | 0.393 |
| Self-reported health status in ASHFS (baseline) |  |  |
| Very good | Ref |  |
| Good | **-0.63 (-1.23, -0.04)** | **0.036** |
| Average/Poor/Very Poor | **-1.58 (-2.36, -0.81)** | **<0.001** |
| **Mental component summary** |  |  |
| BMI-z score in ASHFS |  |  |
| Change in BMI-z score from ASHFS to CDAH-1 |  |  |
| Change in BMI-z score from CDAH-1 to CDAH-2/-3 | **-0.74 (-1.29, -0.19)** | **0.009** |
| Age in CDAH-2/-3 (mid-adulthood), year | -0.02 (-0.09, 0.05) | 0.589 |
| Gender |  |  |
| Male | Ref |  |
| Female | **-1.00 (-1.63, -0.38)** | **0.002** |
| Self-reported health status in ASHFS (baseline) |  |  |
| Very good | Ref |  |
| Good | **-1.07 (-1.76, -0.37)** | **0.003** |
| Average/Poor/Very Poor | **-1.06 (-1.95, -0.18)** | **0.018** |
| **Health state utilities** |  |  |
| BMI-z score in ASHFS | **-0.007 (-0.014, -0.001)** | **0.017** |
| Change in BMI-z score from ASHFS to CDAH-1 | **-0.006 (-0.013, 0)** | **0.048** |
| Change in BMI-z score from CDAH-1 to CDAH-2/-3 | **-0.014 (-0.023, -0.005)** | **0.003** |
| Age in CDAH-2/-3 (mid-adulthood), year | **0.008 (0.007, 0.009)** | **<0.001** |
| Gender |  |  |
| Male | Ref |  |
| Female | -0.010 (-0.020, 0) | 0.052 |
| Self-reported health status in ASHFS (baseline) |  |  |
| Very good | Ref |  |
| Good | **-0.015 (-0.026, -0.003)** | **0.011** |
| Average/Poor/Very Poor | **-0.026 (-0.041, -0.012)** | **<0.001** |

ASHFS, Australian Schools Health and Fitness Survey; BMI: body mass index; CDAH, Childhood Determinants of Adult Health study; CI: confidence interval; Ref, reference group.

Values in bold are statistically significant result.

**Table S4** Distribution of health-related quality of life and health state utilities in mid-adulthood, by subgroup of BMI-z score from childhood to mid-adulthood

| Subgroup of BMI-z score from childhood to mid-adulthood (N=1,069)* | n (%) | Mean (SD) | | |
| --- | --- | --- | --- | --- |
|  |  | Physical HRQoL | Mental HRQoL | Health State Utility |
| Lower BMI change group | 1,029 (45.7) | 52.1 (7.5) | 50.3 (8.9) | 0.764 (0.126) |
| Highest BMI increase group | 40 (1.8) | 47.7(7.8) | 45.5 (10.7) | 0.721 (0.121) |

HRQoL: health-related quality of life; SD: standard deviation.

*Participants were defined as having the highest increase in BMI over the life course (highest BMI increase group) if their increase in BMI-z score was persistently more than one standard deviation (SD) from ASHFS (baseline, childhood) to CDAH-1 (first adult follow-up, young adulthood) and from CDAH-1 to CDAH-2/-3 (mid-adulthood). Participants were defined as the lower BMI change group if their BMI-z score and change in BMI-z score over the life course were persistently within one SD.

**Table S5** The association of subgroup of BMI-z score from childhood to mid-adulthood with health-related quality of life and health state utilities in mid-adulthood

| Subgroup of BMI-z score from childhood to mid-adulthood (N=1,069)* | Physical HRQoL | |  | Mental HRQoL | |  | Health State Utilities | |
| --- | --- | --- | --- | --- | --- | --- | --- | --- |
|  | Unadjusted model | Adjusted Model 1^#^ |  | Unadjusted model | Adjusted Model 1^#^ |  | Unadjusted model | Adjusted Model 1^#^ |
|  | β (95% CI) | β (95% CI) |  | β (95% CI) | β (95% CI) |  | β (95% CI) | β (95% CI) |
| Lower BMI change group | Ref | Ref |  | Ref | Ref |  | Ref | Ref |
| Highest BMI increase group | **-4.26 (-6.65, -1.87)** | **-4.41 (-6.76, -2.06)** |  | **-4.08 (-6.84, -1.32)** | **-3.75 (-6.37, -1.12)** |  | **-0.043 (-0.083, -0.004)** | **-0.045 (-0.083, -0.006)** |

HRQoL: health-related quality of life; CI: confidence interval.

*Participants were defined as having the highest increase in BMI over the life course (highest BMI increase group) if their increase in BMI-z score was persistently more than one standard deviation (SD) from ASHFS (baseline, childhood) to CDAH-1 (first adult follow-up, young adulthood) and from CDAH-1 to CDAH-2/-3 (mid-adulthood). Participants were defined as the lower BMI change group if their BMI-z score and change in BMI-z score over the life course were persistently within one SD.

^#^Adjusted for mid-adulthood age, sex, and self-reported health status at baseline.
